# The influence of tool quality on the machining of additive manufactured and powder metallurgy titanium alloys

**DOI:** 10.1177/09544054221080033

**Published:** 2022-03-01

**Authors:** Junhui Ma, Tanny Tran, Olufisayo A. Gali, Reza A. Riahi

**Affiliations:** Department of Mechanical, Automotive and Materials Engineering, University of Windsor, Windsor, ON, Canada

**Keywords:** Additive manufacturing, powder metallurgy, titanium alloy, drilling, carbide

## Abstract

This study was carried out to investigate the impact the quality of the drill bits has on the machining behavior of additive manufacturing (AM) and powder metallurgy (PM) titanium alloys. Therefore, commercially available drill bits which typically reflect two extremes of drill bit quality were selected. The performance of coated carbide twist drills, typically recommended for the drilling of wrought titanium alloys was compared with that of high-speed steel (HSS) drills. The average torque value, specific cutting energy (SCE), and tool wear were used to evaluate the drilling performance of AM and PM titanium alloys. The results of drilling tests revealed the application of the coated carbide drill resulted in lower torque and SCE values, less flank wear, and lower build-up-edge (BUE) compared with the uncoated HSS drill bits for AM fabricated titanium alloys. However, the carbide drill appeared to offer negligible improvement over the uncoated HSS drill when employed with the PM fabricated titanium alloy. In spite of the improvement in the drilling performance offered by the carbide drills for the AM titanium alloy, TiB intermetallic particles (part of the AM titanium microstructure) contributed to the damage of the coated carbide drill which would limit the drill lifetime.

## Introduction

Titanium alloys are widely used in the aerospace industry and as surgical implants in the medical industry due to their excellent properties such as high fracture resistance, corrosion resistance, and capability to operate at higher temperatures.^1^ However, difficulties are found in the application of titanium alloys due to the difficulty and the high intrinsic cost of machining these alloys. This hard-to-machine property is related to the high chemical reactivity and low thermal conductivity of titanium alloys, leading to an inclination of the titanium workpiece to weld onto the cutting edge of the tools during their machining. The strong adhesion tendency and the low work hardening result in a high wear rate as well as limits the extensive application of titanium alloys.^2,3^ Thus, cost reduction and improved machinability have become the main driving force in the producing and processing of titanium alloys.

Additive manufacturing (AM) and powder metallurgy (PM) technologies provide viable methods to reduce costs in titanium alloy production. This is due to the possibility of reducing the manufacturing steps in fabrication. The main advantages of the two technologies are the ability to build components with three-dimensional complicated geometry shapes through AM processing and near-net-shape components through PM processing. PM processing is characterized by the near finish structure from the compaction of the metal powders, followed by sintering or hot compression.^4,5^ The mechanical properties of titanium alloys fabricated through PM are mainly dependent on their porous microstructure; thus, lower tensile strength and fatigue resistance are obtained compared to ingot metallurgy titanium alloys as their high porosity results in easy crack propagation.^1^ However, the high compressive properties of PM titanium composites containing in-situ whiskers have been reported.^6^ AM technology is characterized by its layer-by-layer manufacturing method, also widely known as 3D printing, providing metallic components with improved accuracy and surface finish with less set-ups.^7,8^ AM fabrication methods are classified into a powder bed system, powder feed system, as well as a wire feed system. During the manufacturing process, AM components have a complicated thermal process, including direction heat extraction, repeated melting, and rapid solidification, leading to different mechanical properties from the conventional process.^9^ Even so, AM alloys have been demonstrated to possess comparable mechanical properties to the components fabricated through the conventional process; however, processing defects including micro-porosity and surface finish could influence the tensile strength and fatigue properties of AM productions.^9,10^

The application of PM and AM technologies in titanium alloys has generally increased due to the significant cost reduction. In like manner, so has research into the processing of alloys fabricated through these technologies to obtain optimal mechanical properties. Tharmaraj and Davidson^11^ reported a method of selectively heating the stress accumulated location of PM aluminum-titanium composites, leading to reduced pores. Jeong et al.^12^ noted that the compressive yield strength of in-situ processed PM titanium composites with the reinforcement of TiB particles was higher than that of Ti-6Al-4V alloy at ambient temperature. Other researches have shown that fully designed PM titanium alloys can obtain comparable fatigue strength and tensile strength by reducing the aspect ratio of alpha phase and refinement of alpha grain.^13,14^ It has been reported that with the addition of TiC particles in PM composite steels, the increase in the stress ratio value was obtained.^15^ In addition, improved wear resistance, specific strength, and fatigue strength for titanium alloys have been reported for alloys fabricated through AM technology by adding ceramic reinforcements, such as SiC, WC, and TiB_2_.^16,17^A study of the mechanical properties of AM titanium alloys has demonstrated that the introduction of a small amount of boron can react with titanium to produce needle-shaped TiB intermetallic particles within the titanium matrix, which aid to greatly improve the fatigue strength.^18^ This was attributed to the good cohesion of the TiB particles to the matrix, as well as their ability to hinder the crack propagation during fatigue testing.^19^

Even though titanium alloys fabricated through PM and AM methods possess the near-fine-shape, further precise machining procedures are still necessary. Generally, cutting tools with protective coatings have been demonstrated to improve the machining performance of metals by slowing down wear processes and prolonging the tool life.^20^ Coated carbide drills have been reported to produce a reduced progression of flank wear growth than uncoated carbide tools in turning wrought tool steel.^21^ However, the machining of PM and AM alloys is more complicated in comparison to the machining of cast or wrought alloys.^22^ The machining process of PM and AM alloys is abrasive in nature and is influenced by the processing parameters, alloying elements, density variations, porosity, and composition of these fabricated alloys. Abduljabbar^23^ noted an increase in machinability of PM 316L stainless steel as the porosity increased. However, Agapiou et al.^24^ reported an opposite trend while machining a PM 304L stainless steel. Andersson and Berg^25^ have reported that with a MnS additive, the machinability of chromium-alloyed PM steels was enhanced in terms of increasing tool life. The research into the machining of PM metal steels appears to be steadily increasing. However, despite the increase in the application of AM and PM titanium alloys, literature on the machining of these alloys is limited.

Previous work^26^ investigated the machinability of AM and PM manufactured titanium alloys and revealed lower torque values as well as lower BUE adhered to the cutting edges of the drills used both associated with the PM alloys. The lower torque values and BUE for the PM titanium alloys were associated with the highly porous microstructure of the PM alloy. While the higher torque values and BUE of the AM titanium alloys were related to the TiB intermetallic particles within the AM titanium microstructure. This study was performed to investigate the influence the quality of the drill bit employed has on the machinability of titanium alloys fabricated through AM and PM technologies. High-quality drill bits in the form of coated carbide drill bits, typically recommended for the drilling of wrought titanium alloys, were selected and compared with uncoated HSS drill bits to reflect the two extremes of drill bit quality. These drill bits were readily commercially available, and the results compared with those from drilling a commercially available wrought titanium alloy to obtain results that are readily transferable to the industry. Torque values generated during the machining process and wear on the cutting edge of the drilling bits measured to evaluate the drills performance, specific cutting energy (SCE) was calculated to aid the evaluation of the drills, by comparing the energy consumed to remove material for each test. Extensive research exists on the influence of drill tool materials and coatings on the machinability of wrought titanium alloys. However, research is limited on determining whether the tools identified as suitable for wrought titanium alloys perform similarly with PM and AM fabricated titanium alloys. The novelty of this research is in the examination of the influence of commercially readily available drill tools on the machinability of PM and AM titanium alloys, which possess varying microstructures. Plasma transferred arc freeform fabrication (PTA-SFFF), a unique form of directed energy deposition was employed to fabricate the titanium alloy, which possessed TiB intermetallic particles as part of the microstructure of the AM titanium alloy. Research showcasing the machining of PM and AM titanium alloys remains limited.

## Experimental procedure

**Figure 1.**
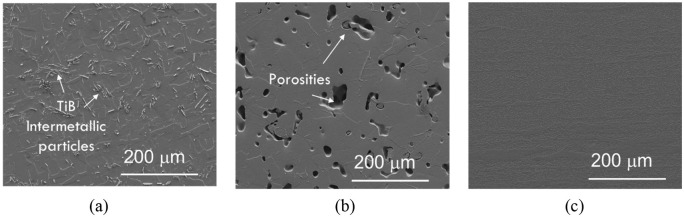
SEM images of: (a) AM titanium alloy showing the TiB intermetallic particles within its microstructure, (b) the porous microstructure of PM titanium alloy, and (c) microstructure of wrought Ti6Al4V.

Titanium alloy blocks with dimensions 300 ×60×13 (L ×W×T) mm fabricated through AM and PM methods were selected as the workpiece. The PM titanium was cold pressed and sintered, with a composition of 99.03% titanium, 0.32% silicon, 0.24% iron, and 0.18% cobalt. The AM titanium was developed by plasma transferred arc solid freeform fabrication (PTA-SFFF), a form of directed energy deposition. The titanium powder was allowed to flow into a plasma beam, and the fabricated alloy was developed by building layer by layer. The AM fabricated alloy had a composition of 97.3% titanium, 0.82% aluminum, 0.63% boron, 0.37% silicon, and 0.2% iron. Further process parameters are the confidential proprietary information of the manufacturer. A wrought Ti-6Al-4V was also considered as a basis of comparison of the machinability of AM and PM alloys. The wrought Ti-6Al-4V was chosen because it is one of the more commonly used titanium alloys, and the AM and PM titanium alloys had been thought of as a possible replacement. The microstructures of the AM, PM, and wrought titanium alloys are displayed in the scanning electron microscope (SEM) images presented in Figure 1. The mechanical properties of these alloys are listed in Table 1.

**Table 1.** Mechanical properties for AM, PM, and wrought titanium.^14^

	Density(g/cm^3^)	Hardness(HRB)	Ultimate tensilestrength (MPa)	Yield strength(MPa)	Compressiveyield strength (MPa)	Porositypercentage (%)
PM titanium	4.11	79	455	360	395	6.35
AM titanium	4.46	97	551	447	588	0.25
Wrought titanium	4.43	109	950	827	970	

**Figure 2.**
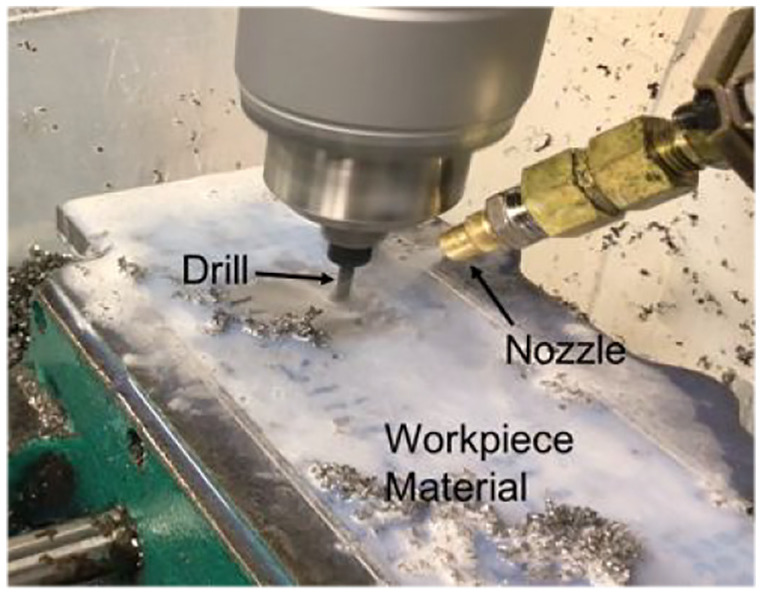
Experimental set-up for the drilling machine.

High-speed steel (HSS) twist drills of 4 mm-diameter manufactured by Viking Drill & Tool Co and M.A. Ford 4 mm-diameter carbide (WC) twist drills were used as the cutting tools. The high-speed steel drills were uncoated, while the carbide drills possessed a physical vapor deposition (PVD) AlTiN coating with a 2 μm thickness. The drill bits consisted of two flutes with a high helix and a split point angle of 135°. All drill bits were cleaned ultrasonically with hexane before the drilling tests, and a single individual drill bit was used for each drilling test schedule. Each test schedule comprised of drilling a set of 60 holes, each to a depth of 5.5 mm. The test cycles were repeated at least twice to ensure the repeatability of the tests. Drilling tests were carried out on a modified computer numerically controlled (CNC) vertical drill press with a maximum power of 1.491 kW and a variable rotation speed within the range of 100–5000 rpm.Tests were carried out at a constant spindle speed of 500 rpm with a feed rate of 0.05 mm/rev. Coolants were supplied through a nozzle under the flooding cooling conditions. An image of the drilling setup is provided in Figure 2. The coolant was comprised of an oil-in-water emulsion.

The torque values generated during each drilling test schedule were recorded, and the average torque values were calculated. The specific cutting energy (SCE) was also calculated. The specific cutting energy (SCE), which is defined as the total energy input (Power) divided by the material removal rate (MRR), was then calculated through equation (1). The Power was calculated using equation (2), where *T* is the average torque value (N m) and *N* is the spindle speed (rpm). The drill bits were then ultrasonically cleaned with hexane after the drilling tests and examined using an environmental scanning electron microscope (SEM) under a high vacuum to investigate the damage to the cutting edge of the drills.

(1) $$\mathrm{SCE}=\frac{\mathrm{Power}\;\left(\frac{J}{s}\right)}{\mathrm{MRR}\;\left(\frac{mm^{3}}{s}\right)}$$

(2) $$\mathrm{Power}=\frac{\left(\pi \times T\right)\times N}{30}$$

## Experiment result

### Average torque value and specific cutting energy (SCE)

**Figure 3.**
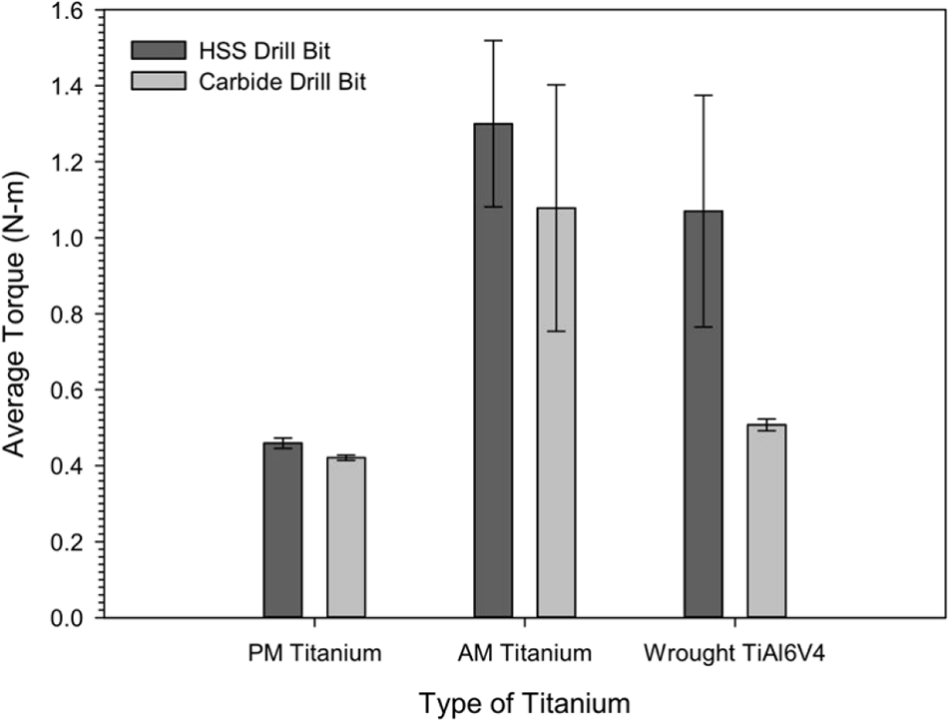
The average torque value for AM and PM titanium alloys and wrought Ti6Al4V machined with the uncoated HSS and coated carbide drills.

The average torque values after a drilling schedule of 60 holes for both the AM and PM titanium alloys with the coated and uncoated drills are displayed in Figure 3. The graph shows that the AM titanium displayed the highest average torque values while the PM titanium possessed the lowest. However, the torque performance of the wrought Ti6Al4V compared with those for the PM and AM titanium alloys were depended on the drill material used. A comparison of the average torque for the PM titanium showed similar values for the uncoated HSS (0.46 ± 0.01 N m) and coated carbide (0.42 ± 0.01 N m) drilling bits. However, the average torque values for the AM titanium showed a higher torque value with the HSS drilling bit (1.30 ± 0.22 N m), than that observed with carbide drilling bit (1.08 ± 0.32 N m). The difference in torque values was observed with the drilling of the wrought Ti6Al4V, with HSS drills displaying a torque value of 1.07 ± 0.31 Nm, while the torque value with the carbide drill bit (0.51 ± 0.02 Nm) was about half of that for the uncoated HSS drill.

Consequently, during machining with the HSS drill, torque values were comparable for the wrought Ti6Al4V and the AM titanium alloy. However, with the coated carbide drill, the torque values of the wrought Ti6Al4V and the PM titanium were similar. It is interesting to note that the torque value associated with the machining of PM titanium with the uncoated HSS drill was still less than half the torque of the AM titanium machined with the coated carbide drill.

**Figure 4.**
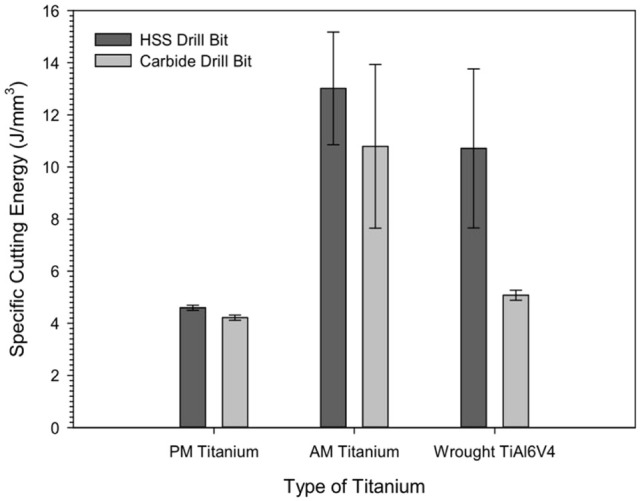
The variation of specific cutting energy (SCE) for AM and PM titanium alloys machined with the uncoated HSS and coated carbide drills.

The specific cutting energy was calculated to evaluate the efficiency of the drilling process of the AM and PM titanium alloys. Lower SCE values are typically associated with the lower energy consumption during the drilling process. Figure 4 displays the SCE variation for AM, PM, and wrought titanium machined with the different drill bits. The graphs showed the lowest SCE values for PM titanium with both HSS (4.59 ± 0.10 J/mm^3^) and carbide (4.21 ± 0.10 J/mm^3^) dill bits. This would indicate that the lowest cutting energy was consumed while machining the PM titanium with the carbide and HSS drills. The AM titanium had a higher SCE value with the use of the HSS drill bit (13.01 ± 2.16 J/mm^3^) than with the carbide drill bit at 10.79 ± 3.13 J/mm^3^. Hence, a reduction in the specific cutting energy was observed by substituting the uncoated HSS drill with the coated carbide drills for the AM titanium. A comparison of the SCE values showed that the wrought Ti6Al4V possessed an SCE value closer to the AM titanium when the HSS drill was employed, although the SCE value for the AM titanium was slightly higher. The SCE value of the wrought Ti6Al4V with the coated carbide drill (5.08 ± 0.19 J/mm^3^) was more in the range of the SCE value of both drills used on PM titanium.

Thus, it could be said that with regards to the torque and SCE, the machining of AM titanium despite the drill used, the uncoated HSS and coated carbide drills, was comparable to the machining of the wrought Ti6Al4V when the HSS drills are employed. The machinability of the AM with both the HSS and coated carbide drills was comparable to the lower machinability of the wrought titanium with the HSS drills. However, the machining of PM titanium with both the uncoated and coated drills is similar to the machining of wrought Ti6Al4V when coated carbide drills are employed.

### Characterization of drill bit

**Figure 5.**
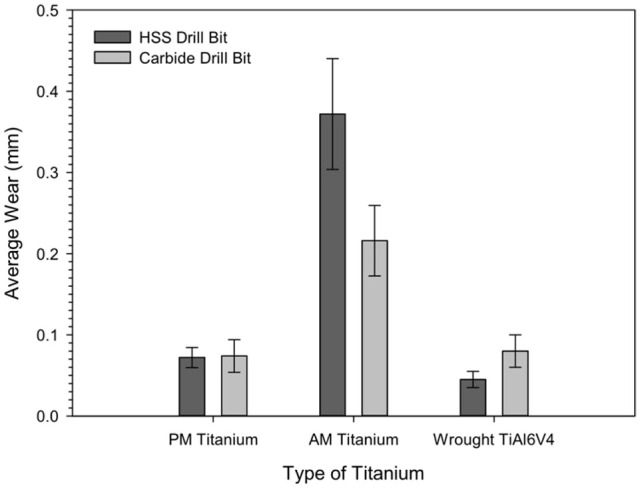
The average flank wear on the uncoated HSS and coated carbide drills after a drilling schedule of 60 holes on AM and PM titanium alloys and wrought Ti6Al4V.

Flank wear damage to the cutting edge was measured for the drills used to machine both the AM and PM titanium alloys after the drilling cycles and is graphically displayed in Figure 5. Drills used on AM titanium possessed generally higher average wear values, the HSS drill displayed more wear at 0.37 ± 0.07 mm than the carbide drill (0.22 ± 0.04 mm). The average wear values on the cutting edge of the drill bits used on PM titanium were comparable for the HSS and coated carbide drills, at 0.072 ± 0.01 and 0.074 ± 0.02 mm, respectively. Wear on the drills used with the AM titanium was generally 4.5 times higher than the wear on the drills used with the PM titanium when HSS drills were used and three times higher when the carbide drills were employed. In comparison, the wear on the HSS drills used on the wrought Ti6Al4V (0.045 ± 0.01 mm) was less than that of the HSS drills used with the AM and PM titanium. However, while the wear on the carbide drills used with the wrought Ti6Al4V (0.080 ± 0.02 mm) was much less than the wear induced by the AM titanium, it was comparable with that related to the PM titanium. It is interesting to note that while wear was observed to reduce with the application of the coated carbide drills for the AM titanium, the reverse was noted for the wrought Ti6Al4V. Regardless, the wear on both the HSS and carbide drill due to the wrought Ti6Al4V was still about 4.5 times lower than that of the AM titanium.

**Figure 6.**
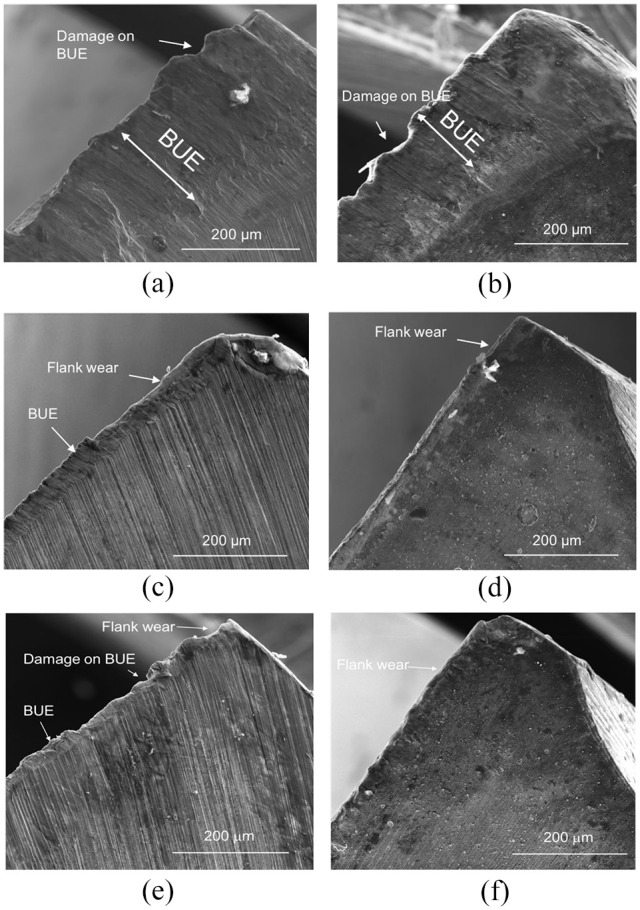
Secondary electron microscope (SEM) images of the cutting edge of drills after drilling AM titanium with: (a) HSS drill and (b) coated carbide drill, on PM titanium with (c) HSS drill and (d) coated carbide drill, and on wrought Ti6Al4V with (e) HSS drill and (f) coated carbide drill.

**Figure 7.**
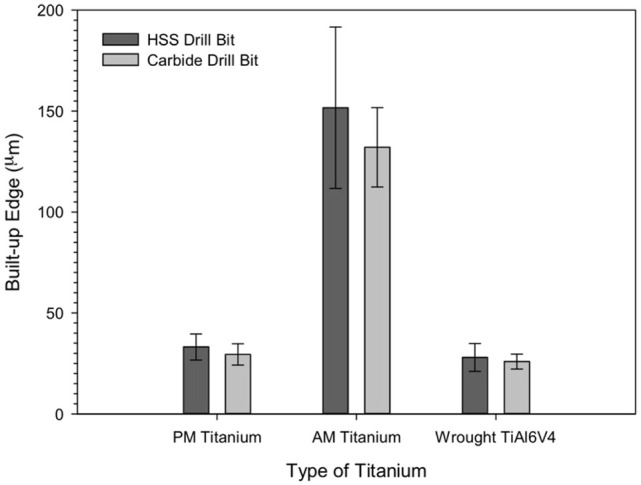
Build-up-edge thickness on the cutting edge at HSS and carbide drill bits after drilling on AM and PM titanium alloys and wrought Ti6Al4V.

The HSS and coated carbide drills were examined, and the SEM images of the cutting edges are displayed in Figure 6. The analysis revealed that the cutting edge of the drill bits used on AM titanium (Figure 6(a) and (b)) was majorly covered with titanium adhesion build-up (BUE). Damage to the accumulation of BUE on the HSS and carbide drills in the form of fracture and chipping of the BUE was noted. This type of damage to the BUE could be caused by the breaking-off of the BUE from the cutting edge during the drilling process and would determine the BUE thickness at each point of drilling. The BUE covering the HSS and carbide drills (Figure 6(c) and (d)) employed to machine the PM titanium displayed little or no damage to the build-up. The BUE accumulation due to machining the PM titanium appeared to be uniform across the cutting edge and less than the BUE accumulation from machining the AM titanium. In comparison, the BUE on the HSS drill used to machine the wrought Ti6Al4V possessed similar damage due to the fracture and chipping as the BUE from the AM titanium. However, the BUE accumulated on the carbide drill from interaction with the wrought Ti6Al4V was not as uniform as the BUE induced from the PM titanium. There was slight damage along the cutting edge of the carbide drill, likely from chipping during the drilling of the wrought Ti6Al4V alloy. The thickness of the BUE observed on the cutting edge after 60 holes was measured, and the graphical representation is presented in Figure 7. It was detected that the BUE on the HSS drills after drilling the AM titanium was thicker at 151.66 ± 39.93 µm than the BUE on the coated carbide drill (132.06 ± 19.66 µm). BUE was much less on the drills employed with the PM titanium; however, the BUE on the HSS drills (33.16 ± 6.46 µm) was thicker than the BUE on the carbide drills (29.46 ± 5.29 µm). Thus, the carbide drill displayed a lower BUE thickness than the HSS drill for both the AM and PM titanium. Interestingly, the wrought Ti6Al4V not only displayed the lowest thickness of the BUE for both the HSS and carbide drills (27.96 ± 6.87 and 25.93 ± 3.69 µm, respectively), the BUE observed on both drills were comparable.

## Discussion

The previous examination^26^ into the machinability of AM and PM titanium alloys revealed that the fabrication process possessed a distinct influence on the machinability of titanium alloys. The higher machinability, in terms of lower torque and SCE values, of the PM titanium, was associated with the porous microstructure of these alloys, while the lower machinability, in the form of higher torque values, of AM titanium, was related to the TiB intermetallic particles within its microstructure. This study aimed to investigate the influence of the drill on the machinability of AM and PM fabricated titanium alloys. A comparison of the torque and SCE values and the wear results revealed similar values for the uncoated HSS drill and the coated carbide drill during the drilling of the PM titanium. There was also little difference in the accumulation of BUE on the cutting edges of the drills. Therefore, it can be said that the coated carbide drills did not offer a significant improvement to the drilling performance of PM titanium alloys. This could be related to the highly porous microstructure of the PM alloy (Figure 1(b)) induced during the fabrication process. The porous PM microstructure would lead to lower strength of the PM alloy and less metal-to-metal contact during drilling; therefore, low torque forces would be required with both the HSS and carbide drills. The reduction in metal-to-metal contact during drilling would likely result in similarly low titanium adhesion to the drill and in turn the similar machining performance of both drills. Thus, the machinability of PM titanium alloys would be more influenced by the porosity of the microstructure induced by this process, as noted by Abduljabbar^23^ and Agapiou et al.,^24^ than the coated carbide drills.

The SCE values evaluated during the drilling of the AM titanium alloys revealed that lower energy was associated with the carbide drill compared with the HSS drill; in other words, higher productivity could be obtained with the application of the coated carbide drill. Along with lower SCE values, lower average torque, and average wear were realized with the use of carbide drills in place of the HSS drills. The lower torque and less energy consumed during the drilling of the AM titanium using the carbide drills could be related to the lower titanium adhesion on the cutting edge. Thus, the coated carbide drill was better adept at mitigating titanium adhesion to the drill during the drilling of AM titanium. Higher titanium adhesion or BUE to the drill cutting edge has been related to high torque values. Increasing titanium adhesion, an indication of high drilling temperatures, hinders drill rotation, while causing severe damage to the drill, which could all result in seizure during drilling. The carbide drill had little influence on the drilling performance of the PM titanium; however, with the AM titanium, the coated carbide drill plays a distinct role. This could be related to the microstructure of the titanium blocks, as with the AM titanium, high metal-to-metal contact is expected due to its low porosity; therefore, the ability to reduce the adhesion to the drill during the machining is vital for improved machinability. The microstructure also influences the strength of the material, which was higher for the AM titanium than for the PM titanium, and while this would explain the higher torque values for the AM titanium in comparison to the PM titanium, does not necessarily explain the influence of the drill material.

The average torque and SCE assessment have revealed that the AM titanium was more difficult to machine than the wrought Ti6Al4V despite the lower strength and hardness of the AM titanium alloy. The lower machinability of the AM titanium could be related to the TiB intermetallic particles within the AM titanium’s microstructure (Figure 1(a)). These TiB intermetallic particles have been observed to hinder crack propagation within this AM titanium alloy. During fatigue tests, these TiB intermetallic particles have been observed to either arrest or deflect crack propagation. High strains along with severe plastic deformation were also observed to induce a nanocrystalline microstructure, which was noted surrounding propagating cracks.^19^ However, the effect of the carbide drill on the machinability of the AM titanium was similar to that of the wrought Ti6Al4V. This influence of the carbide drills on the machinability of the AM titanium and wrought Ti6Al4V could be related to their non-porous microstructure. The PM titanium displayed better machinability than the wrought Ti6Al4V with the HSS drills; conversely, it possessed similar machinability to the wrought Ti6Al4V with the carbide drills. Thus, in terms of SCE and torque, the introduction of the carbide drill had a greater influence on the wrought Ti6Al4V than on the PM or AM titanium. Generally, carbide drills are characterized by improved machinability due to the lower wear rate they offer. This is the reason coated carbide drills are typically recommended for the drilling of wrought titanium alloys. The difference in the influence of the carbide drill on the three alloys due to the difference in the microstructure could indicate that the optimal drill for each alloy would depend on the fabrication method of the titanium alloy. The porosity of the microstructure of the titanium alloy could mean that the drill type could be irrelevant, however, more research would be required for confirmation as well as determine which coating would lead to a more definite improvement in machinability for the AM titanium alloy.

**Figure 8.**
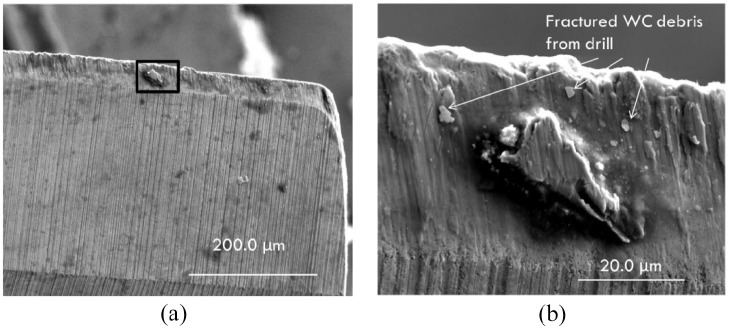
Fractured WC piece on the coated carbide drills after 10 holes drilled on AM titanium alloy under (a) lower and (b) higher magnification.

The coated carbide is typically meant to hinder titanium adhesion to the drill. To understand the BUE accumulation on the carbide drills, they were examined after 10 holes had been machined into the AM titanium. The examination of the cutting edge (Figure 8(a)) under this condition revealed titanium adhesion to the drill. However, closer examination (Figure 8(b)), taken from the boxed region in Figure 8(a), revealed fractured WC pieces on the cutting edge of the carbide drill used on the AM titanium alloy. Consequently, damage to the carbide drill had begun after drilling 10 holes. Damage to the carbide drill would reduce the integrity of the coating, which would contribute to the initiation of adhesion to the cutting edge. The carbide drill’s continued fracture damage during drilling subsequent holes could lead to the tool’s failure. The failure of the coated carbide at portions of the cutting edge could result in the high BUE observed at the end of a 60-hole drilling cycle. Thus, although improved drilling performance was obtained from the coated carbide drills, there were limitations to the performance of these drills due to the damage induced on them during the drilling cycle of the AM titanium. In the case of the wrought Ti6Al4V, the carbide drill also showed evidence of chipping to the drill’s cutting edge; however, this damage to the carbide drill was considerably less in comparison to that induced by the AM titanium.

The WC debris would also aid in the reduction of the torque observed during the drilling of both the wrought Ti6Al4V and AM titanium alloys. Thus, the WC debris could be one of the mechanisms for the lower torques observed with the carbide drills.

**Figure 9.**
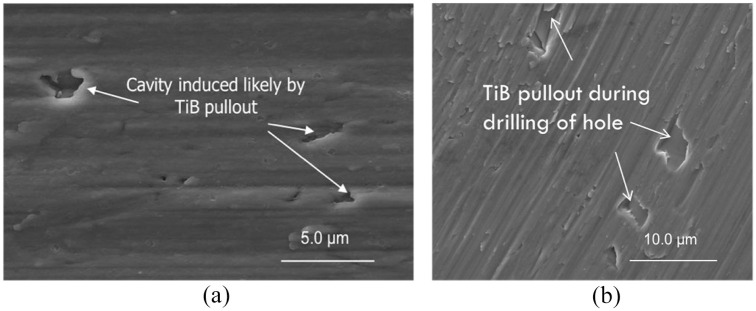
The cavities induced by the pullout of TiB particles observed on the: (a) surface of the drilled wall on AM titanium alloy and (b) surface of chips generated from AM titanium alloy.

**Figure 10.**
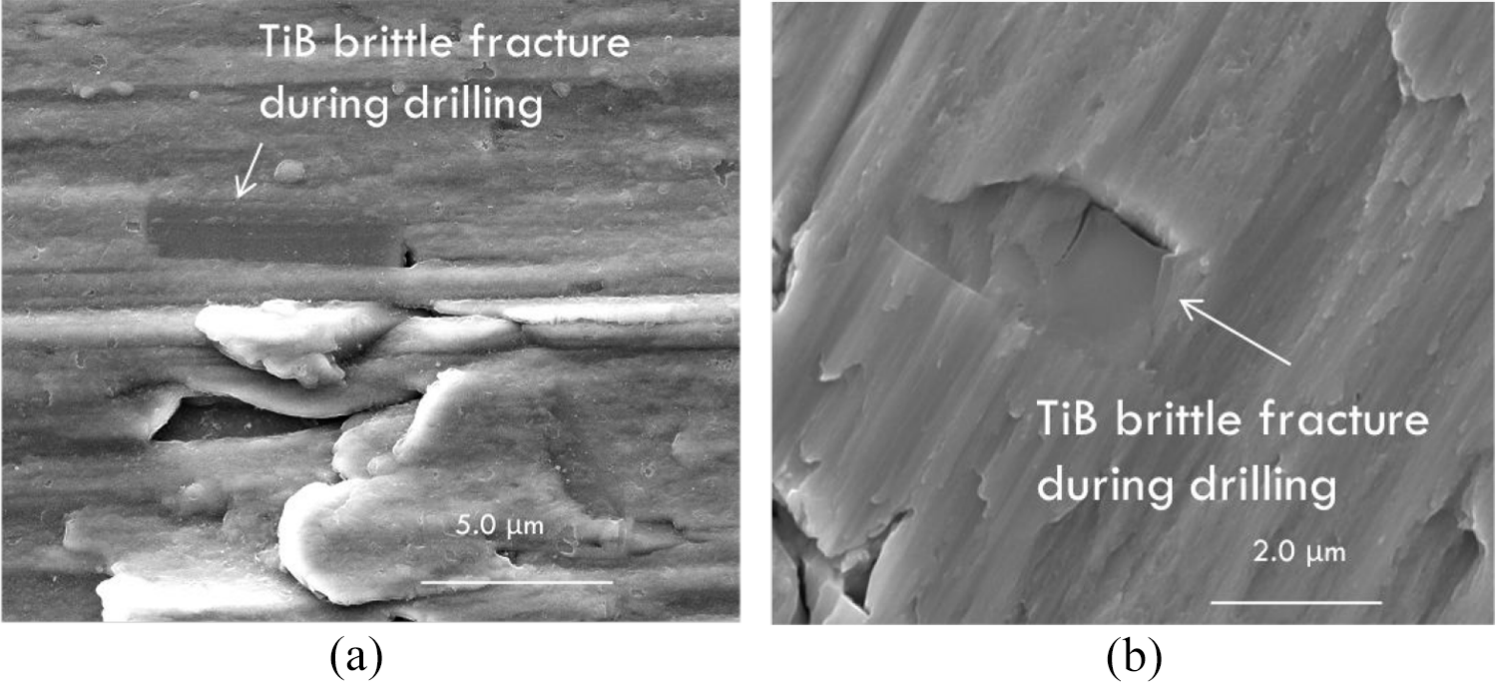
Fractured TiB particles embedded within: (a) the drilled wall surface and (b) chip surface from AM titanium alloy.

The increased damage to the carbide drill during the drilling of the AM titanium alloy could also be related to the TiB intermetallic particles within its microstructure. An examination of the holes drilled into the AM titanium alloy after the 10 holes drilling cycle and the chips developed during this process exposed that the TiB particles had experienced fracture and pullout. Cavities were observed on the drilled hole (Figure 9(a)) and chip surface (Figure 9(b)), which could be formed from the pull-out of the TiB intermetallic particles. Figure 10 displays the brittle fracture of the TiB intermetallic particles embedded within the wall of the drilled AM titanium hole and chip surface. The hard ceramic TiB intermetallic particles require a larger thrust force to cut than the titanium matrix. The TiB particles could be pulled out or sheared due to the increased thrust force during the drilling process.^18^ The distribution of TiB particles leads to higher thrust force during the drilling operations. The higher trust force could result in the fracture of the coated carbide as well as the pullout and fracture of the TiB intermetallic particles. The fracture of the coated carbide could also occur due to the fracture or pullout of the TiB intermetallic particles. The fractured or pullout intermetallic TiB particles debris could damage the carbide drill. Thus, the higher difficulty of machinability of AM titanium alloy could also result from the damage to the drill due to the TiB intermetallic particles. It is thus plausible to infer that if the integrity of the drill material was improved, the damage induced during drilling as a result of the TiB intermetallic particles would be reduced and, the machinability of the AM titanium could be greatly improved.

In summary, while the microstructure of the PM titanium alloy negates the need for the carbide to aid in improving its machinability, the machinability of the AM titanium alloy is improved by the employment of the coated carbide drill. The TiB intermetallic particles within the titanium matrix of the AM titanium led to damage to carbide drills, high BUE accumulation, which resulted in high torque and SCE values. Therefore, the integrity and lifetime of the coated carbide drill during the drilling process could be influenced by the TiB intermetallic particles. It is also noted that the machinability of the PM titanium is generally similar to that of the wrought Ti6Al4V when the Ti6Al4V is machined with a coated carbide drill. Conversely, the machinability of the AM titanium is comparable to that of the wrought Ti6Al4V when the wrought alloy is machined with an uncoated HSS drill.

## Conclusion

This study was carried out to investigate the effect of coated carbide drills on the drilling performance of additive manufacturing (AM) and powder metallurgy (PM) titanium alloys under the same cutting conditions. A series of tests were performed with the application of HSS and coated carbide drills to evaluate their influence on the machinability of the AM and PM titanium alloys. The results indicate as below:

1. The drilling performance of the PM titanium alloys displayed little influence from the employment of the coated carbide drills in place over uncoated HSS drills, which could be related to the porous microstructure of the PM titanium alloy.
2. An improvement in drilling performance was observed with the application of coated carbide drills during the machining of AM titanium alloys. The employment of coated carbide drills resulted in lower torque and SCE values, less wear, and lower BUE compared with machining with uncoated HSS drills.
3. The improvement in performance with the carbide drills could be related to the coated carbide’s ability to reduce titanium adhesion and the accumulation of BUE during the drilling of the AM titanium. However, the TiB intermetallic particles, which are part of the AM microstructure, could damage the carbide drill and limit the drills’ lifetime.
